# Sexual, irritative, and voiding outcomes, following stereotactic body radiation therapy for prostate cancer

**DOI:** 10.1186/s13014-015-0488-3

**Published:** 2015-08-28

**Authors:** Zaker Rana, Robert L. Hong, Mustafa Abugideiri, Donald McRae, George Cernica, Robert Mordkin, Andrew B. Joel, Gregory Bernstein, Nadim M. Nasr

**Affiliations:** Department of Radiation Oncology Medicine, Virginia Hospital Center, 1701 N George Mason Dr, Arlington, VA 22205 USA

## Abstract

**Background:**

Urinary symptoms and sexual dysfunction are the two most common complaints following prostate radiotherapy. The impact of hypofractionated treatment on sexual function, irritative symptoms, and voiding symptoms has not been determined within the same patient population. Here we present our institutional data on sexual function, voiding function, irritative symptoms, and treatment response following SBRT.

**Methods:**

This retrospective analysis includes 102 non-metastatic patients treated with SBRT at a single institution between May 2008 and September 2014. The course of radiotherapy consisted of 36.25 Gy (range 35–40) over five daily fractions. International Prostate Symptom Score (IPSS), Sexual Health Inventory for Men (SHIM), and PSA were recorded at baseline, 1, 3, 6, 9, 12, 18, 24, and 36 months after treatment.

**Results:**

Median patient age was 72 years old with a median follow-up of 4.3 years. Pretreatment IPSS-I score was 5.21, increasing to 6.97 (*p* < .001) after 1 month. The mean IPSS-I score returned close to baseline after 3 months to 5.86 and decreased to below baseline after 2 years to 5.09. At 3 months, 9 months, and 2 years, 47.5, 76.2, and 91.1 % of patients had reached IPSS-I resolution. The mean IPSS-O score prior to treatment was 5.31 and there was an increase in the score to 6.45 (*p* = 0.344) at 1 month. The score remained close to baseline and decreased to 4.00 at 2 years and significantly decreased to 3.74 (*p* = 0.035) at 3 years. 64.4, 82.1, and 96.0 % of patients had IPSS-O resolution by 3 months, 9 months, and 2 years. The mean SHIM score prior to treatment was 13.52 and continually decreased to below baseline a year after treatment to 10.56 (*p* < .001). SHIM score began to improve at 18 months, but was still significantly less than baseline at 12.12 (*p* = .01).

**Conclusions:**

While an increase in AUA/IPSS score initially occurred, all patients resume normal activities immediately following treatment and the AUA/IPSS symptoms improved from baseline. Irittative symptoms take longer to resolve when compared to obstructive voiding symptoms in patients treated with SBRT. Three year PSA response, reported toxicity, erectile function preservation, and urinary function improvement, shows favorable results.

## Background

Stereotactic body radiation therapy (SBRT) allows for precise high dose radiation with minimal exposure to adjacent healthy tissue [1, 2]. Prostate cancer with an estimated low α/β ratio of 1.0–1.7 may be more amenable to a hypofractionated treatment approach via radiosurgical techniques compared to standard fractionated external beam techniques [3]. Given the reduced number of sessions, hypofractionation may be more convenient, especially to patients with limited access to care. Numerous studies have demonstrated the efficacy of several different hypofractionation approaches including 2.5 Gy per fraction to 70 Gy and 3.1 Gy per fraction to 62 Gy [4–13].

Recently, studies have demonstrated the efficacy of more aggressive treatment regimens utilizing stereotactic body radiation therapy (SBRT) techniques up to 10 Gy per fraction to 50 Gy. However, while higher doses result in a lower risk of biochemical failure, they also increase the risk of bladder, rectal and small bowel toxicity [14–20]. There is limited data suggesting a superior treatment for prostate cancer, and treatment is often based on patient preference and a patient’s subsequent health-related quality of life [21].

Lower urinary tract symptoms (LUTS) and sexual dysfunction are common following both surgery and radiation therapy [22]. Obstructive voiding symptoms and irritative symptoms are common LUTS and the prevalence could be as high as 30 % in men older than 70 [23]. Among men, the prevalence of LUTS has been shown to increase with age. However, when comparing prevalence of severe symptoms (an IPSS ranging between 20 and 35) there is no significant difference among various younger age groups [24]. Initial reports suggest that the incidence of irritative and obstructive symptoms resulting from SBRT may be less than brachytherapy and comparable to external radiation therapy [20, 25, 26]. Erectile dysfunction is one of the most concerning toxicities after radiotherapy. Conventionally fractionated external beam radiation therapy (EBRT), brachytherapy, and SBRT all have an affect on erectile function [27–29]. The dysfunction experienced by patients often returns to baseline with time [30].

The goal of our study is to illustrate not only the efficacy of using SBRT as a treatment modality for organ confined prostate cancer, but also to demonstrate that SBRT approach results in acceptable urinary and sexual toxicity. Here we present our institutional data on sexual function, voiding function, irritative symptoms, and treatment response following SBRT.

## Methods

### Study design

This retrospective analysis includes prostate cancer patients treated with SBRT at a single institution on an IRB approved protocol between May 2008 and September 2014. Eligible patients had biopsy-proven newly diagnosed, non-metastatic and untreated prostate cancer. Study endpoints included early and late urinary toxicity, sexual function questionnaire-based measures, and PSA response. Prostate cancer risk stratification followed the standard D’Amico risk stratification (low risk: PSA <10 and Gleason sum of 6 and clinical stage T1c–T2a, intermediate risk: PSA 10–20 or Gleason sum of 7 or clinical stage T2b, and high-risk: PSA >20 or Gleason sum 8–10 or clinical stage T2c/T3).

### Treatment specifics

The CyberKnife (Accuray Inc., Sunnyvale CA) was used to deliver fiducial-based image-guided SBRT. Four gold fiducials were placed in the prostate via a trans-rectal or trans-perineal approach using trans-rectal ultrasound guidance, followed by a non-contrast CT scan in the supine position. Anatomical contours of the prostate, seminal vesicles, rectum, bladder, bladder neck, penile bulb, and femoral heads were generated. The homogeneous planning dose was prescribed to the planning target volume (PTV) that consisted of a volumetric expansion of the prostate by 5 mm, reduced to 3 mm in the posterior direction. The course of radiotherapy consisted of 36.25 Gy (range 35–40 Gy) over five daily fractions. Dose was normalized to the 90 % isodose line in order for the prescription dose to cover at least 95 % of the PTV. Generally speaking, dose volume histogram (DVH) goals for the rectum were such that the V50 % <50 % (i.e., the volume receiving 50 % of the prescribed dose was <50 %), V80 % <20 %, V90 % <10 % and V100 % <5 %. The bladder DVH goals were V50 % <40 % and V100 % <10 %. The femoral head DVH goal was V40 % <5 %. A short course (median 4 months) of neoadjuvant and concurrent androgen deprivation therapy (ADT) was allowed at the discretion of the treating physician.

### Follow-up and analysis

In general, PSAs were obtained at baseline, and prospectively at 3-month post-treatment intervals during the first 2 years and at 6-month intervals thereafter. The PSA relapse definition used was the currently adopted standard of care Phoenix definition (i.e., nadir +2). A benign PSA bounce was called when PSA rose by > 0.2 ng/mL above the post-treatment nadir and subsequently returned to nadir levels or below.

Toxicities were recorded using the Common Terminology Criteria for Adverse Events. American Urological Association/International Prostate Symptom Score (AUA/IPSS) and Sexual Health Inventory for Men (SHIM) were recorded at baseline, 1, 3, 6, 9, 12, 18, 24, and 36 months after treatment. The IPSS includes four questions related to obstructive symptoms (incomplete emptying, intermittency, weak stream, and straining) as well as three questions related to irritative symptoms (frequency, urgency and nocturia). For all the questions except the nocturia question, the responses were grouped into four clinically relevant categories (never, less than half the time, half or more than half the time, and almost always). For the nocturia question, the responses were grouped into four clinically relevant categories (none, 1 time, 2 times and > 3 times). The IPSS obstructive subscore (IPSS-O) has been defined at the sum of the scores for questions one, three, five, and six, while the IPSS irritative subscore (IPSS-I) has been defined as the sum of the scores for questions two, four, and seven [31]. Overall IPSS-I scores range from 0 to 15 and IPSS-O scores range from 0 to 20 with higher scores indicating greater severity [22].

Wilcoxon Signed-Rank Test and chi-square analysis were used to compare AUA/IPSS and SHIM scores to baseline. Binary logistic regression was used in the multivariate analysis to search for possible predicting factors for IPSS-I or IPSS-O improvement. The endpoint for this analysis was an IPSS-I/IPSS-O score at least one point lower than baseline at 3 years post-SBRT. Baseline characteristics including age, race, prostate volume, and baseline ADT use were included as variables in the logistic regression model. Time to IPSS, IPSS-I, and IPSS-O resolution were determined using the Kaplan-Meier method. Continuous variables were expressed using sample medians and ranges.

## Results

From May 2008 to August 2013, 102 patients with a median follow-up of 4.3 years were treated with SBRT. The median patient age was 72 (47–88) years old with 55.6 % of the patients being white, and 26.8 % being black (Table 1). The median prostate volume was 43 cc (14–170.7) and 8.9 % of patients used androgen deprivation therapy. 36.3 % of patients were low risk, 54.9 % of patients were intermediate risk, and 7.8 % of patients were high risk based on the D’Amico classification. 58.4 % of patients had moderate to severe lower urinary tract symptoms (baseline AUA ≥ 8) and 73.7 % of patients had some level of erectile dysfunction (baseline SHIM ≤ 21) prior to treatment.

**Table 1:** Baseline patient and treatment characteristics

		Patients (*N = 101*)
Age (y/o)	Median 72 (47–88)	
	Age ≤ 60	13.9 %
	60 < Age ≤ 70	51.5 %
	Age > 70	34.6 %
Race	White	55.6 %
	Black	26.8 %
	Other	17.6 %
Median prostate volume (cc)	Median: 43 (14.0–170.7) cc	
Risk groups (D’Amico’s)	Low	36.3 %
	Intermediate	54.9 %
	High	7.8 %
ADT		8.9 %
Baseline AUA score	Median: 9.5	
	Mild (1–7):	41.6 %
	Moderate (8–19):	41.6 %
	Severe (20–35):	16.8 %
Baseline SHIM score	Median: 15	
	Severe ED (1–7):	34.2 %
	Moderate ED (8–11):	10.5 %
	Mild-Moderate ED (12–16):	7.9 %
	Mild ED (17–21):	21.1 %
	Normal function (>21):	26.3 %

**Fig. 1:**
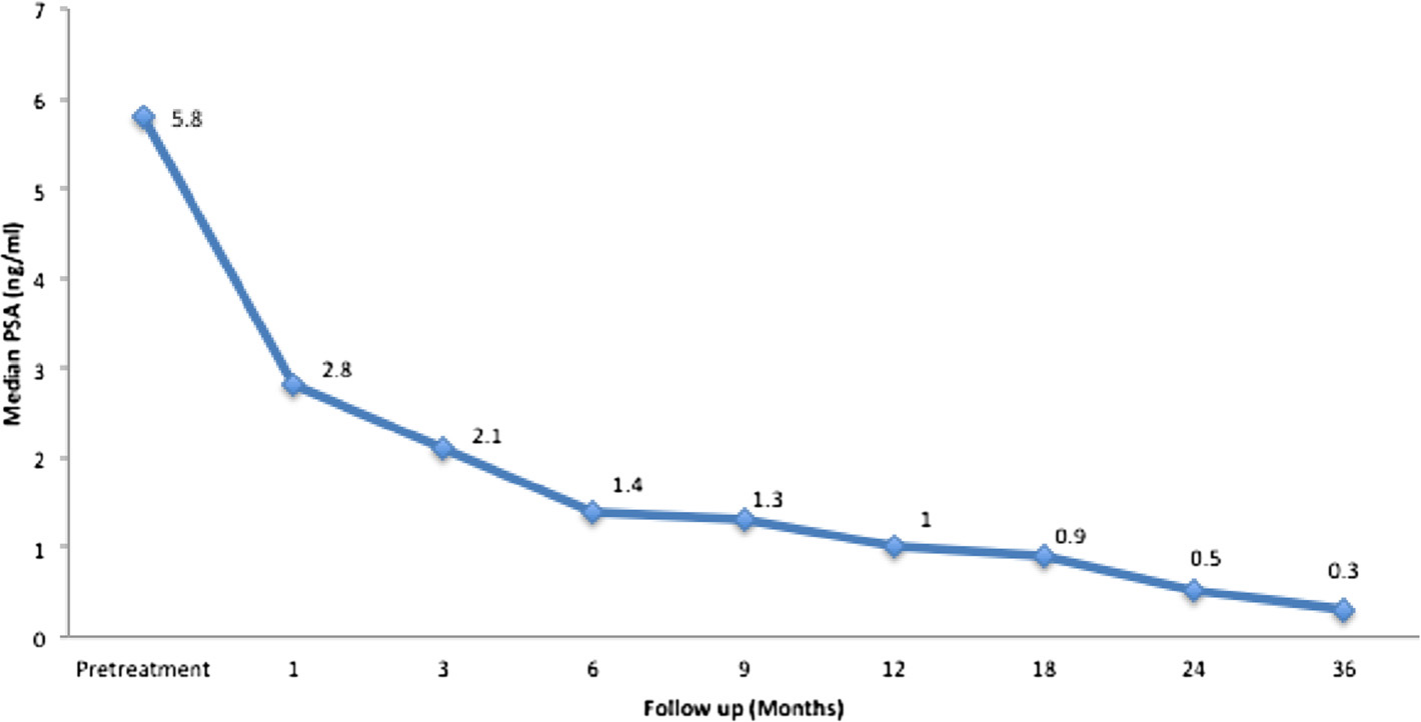
Median PSA for all patients before and after treatment

The median initial PSA was 5.8 ng/ml (Fig. 1). One month after treatment, the median PSA decreased to 2.8 ng/ml. Six months after treatment the PSA was 1.4 ng/ml and then it continued to decrease to 1 ng/ml after 1 year, 0.5 ng/ml after 2 years, and 0.3 ng/ml after 3 years. There were no biochemical failures and benign PSA bounces occurred in 24 % with a median PSA bounce of 0.6 ng/ml.

**Fig. 2:**
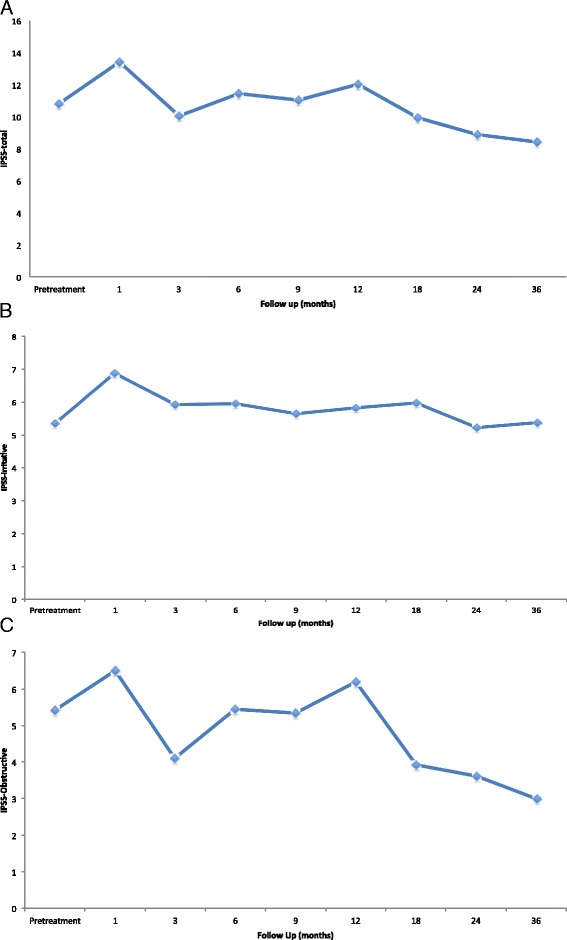
IPSS subscores over the 36 months of follow-up **a** Mean IPSS-total score for all patients. **b** Mean IPSS-Irritative score for all patients. **c** Mean IPSS-obstructive score for all patients

**Fig. 3:**
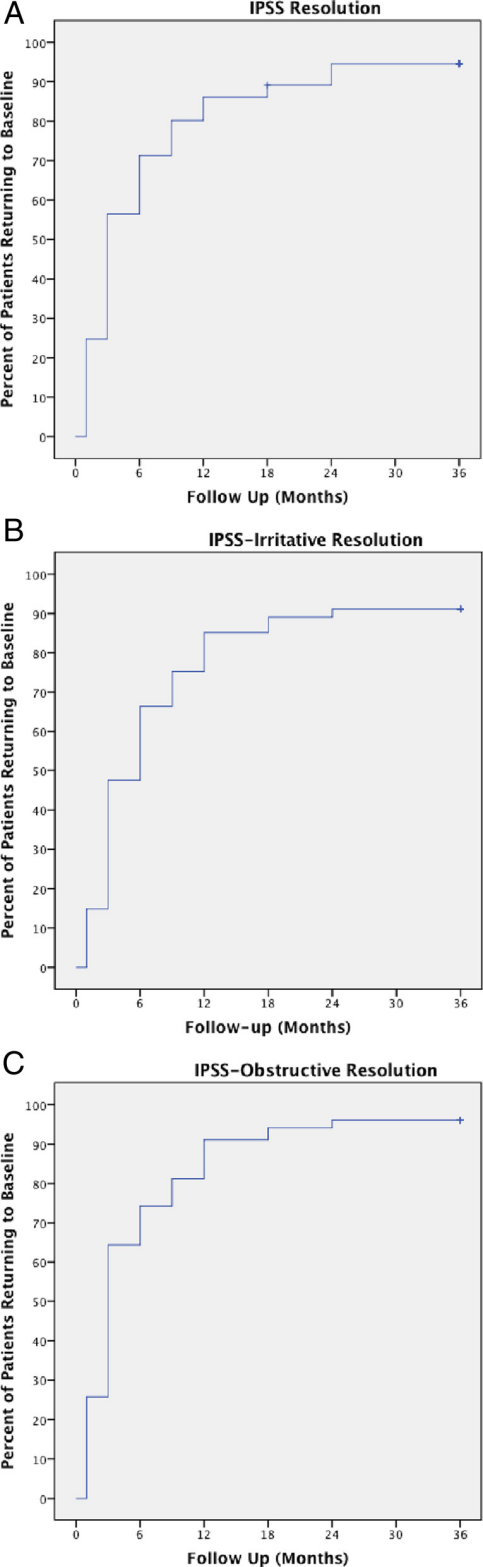
IPSS resolution time **a** Total IPSS resolution time (return to within two points of baseline score) **b** Total IPSS-irritative resolution time (return to within one point of baseline score) **c** Total IPSS-obstructive resolution time (return to within one point of baseline score

The mean AUA/IPSS score for all patients was 10.48 at baseline, which corresponds with mild-to-moderate lower urinary tract symptoms (Table 2) (Fig. 2a). After 1 month, AUA/IPSS significantly increased to 13.38 (*p* < .001). The scores returned to baseline by 3 months at 10.64, slightly worsened at 1 year to 11.11, but then decreased below baseline by 2 years with a mean score of 9.00. The median time to IPSS resolution was 3 months with 56.4 % of patients returning to baseline by this time (Fig. 3a). By 9 months, 80.2 % of patients had reached IPSS resolution and by 2 years 95 % of patients treated had resolved. Pretreatment IPSS-I score was 5.21, significantly increasing to 6.97 (*p* < .001) after 1 month (Fig. 2b). The mean IPSS-I score returned close to baseline after 3 months to 5.86 and decreased to below baseline after 2 years to 5.09. At 3 months, 47.5 % had reached IPSS-I resolution, at 9 months 76.2 % had reached resolution, and at 2 years 91.1 % of patients treated reached IPSS-I resolution (Fig. 3b). The mean IPSS-O score prior to treatment was 5.31 and there was a non-statistically significant increase in the score to 6.45 (*p* = 0.344) at 1 month (Fig. 2c). The score remained close to baseline and decreased to 4.00 at 2 years and significantly decreased to 3.74 (*p* = 0.035) at 3 years. 64.4 % of patients had IPSS-O resolution by 3 months, 82.1 % by 9 months, and 96.0 % by 2 years (Fig. 3c). Total IPSS score and irritative score decreased after an initial spike at 3 months, however all IPSS sub-scores had a minor but not statistically significant increase at 12 months.

**Table 2:** Changes in mean IPSS-total, IPSS-irritative, and IPSS-obstructive, from baseline following SBRT for prostate cancer

	Pre	1 mo	3 mo	6 mo	9 mo	12 mo	18 mo	24 mo	36 mo
IPSS-Total									
Mean	10.48	13.38	10.64	10.20	10.33	11.11	10.59	9.00	8.55
Mean difference		2.90	0.16	−0.28	−0.15	0.63	0.11	−1.48	−1.94
*p*-value		< .001	1	1	1	1	1	1	.478
IPSS-irritative									
Mean	5.21	6.97	5.86	5.45	5.51	5.77	5.41	5.09	4.81
Mean difference		1.76	0.65	0.24	0.30	0.56	0.20	−0.12	−0.40
*p*-value		< .001	1	1	1	1	1	1	1
IPSS-obstructive									
Mean	5.31	6.45	4.92	4.82	4.85	5.31	5.20	4.00	3.74
Mean difference		1.14	−0.39	−0.49	−0.46	0.00	−0.10	−1.31	−1.56
*p*-value		0.344	1	1	1	1	1	0.134	.035

**Fig. 4:**
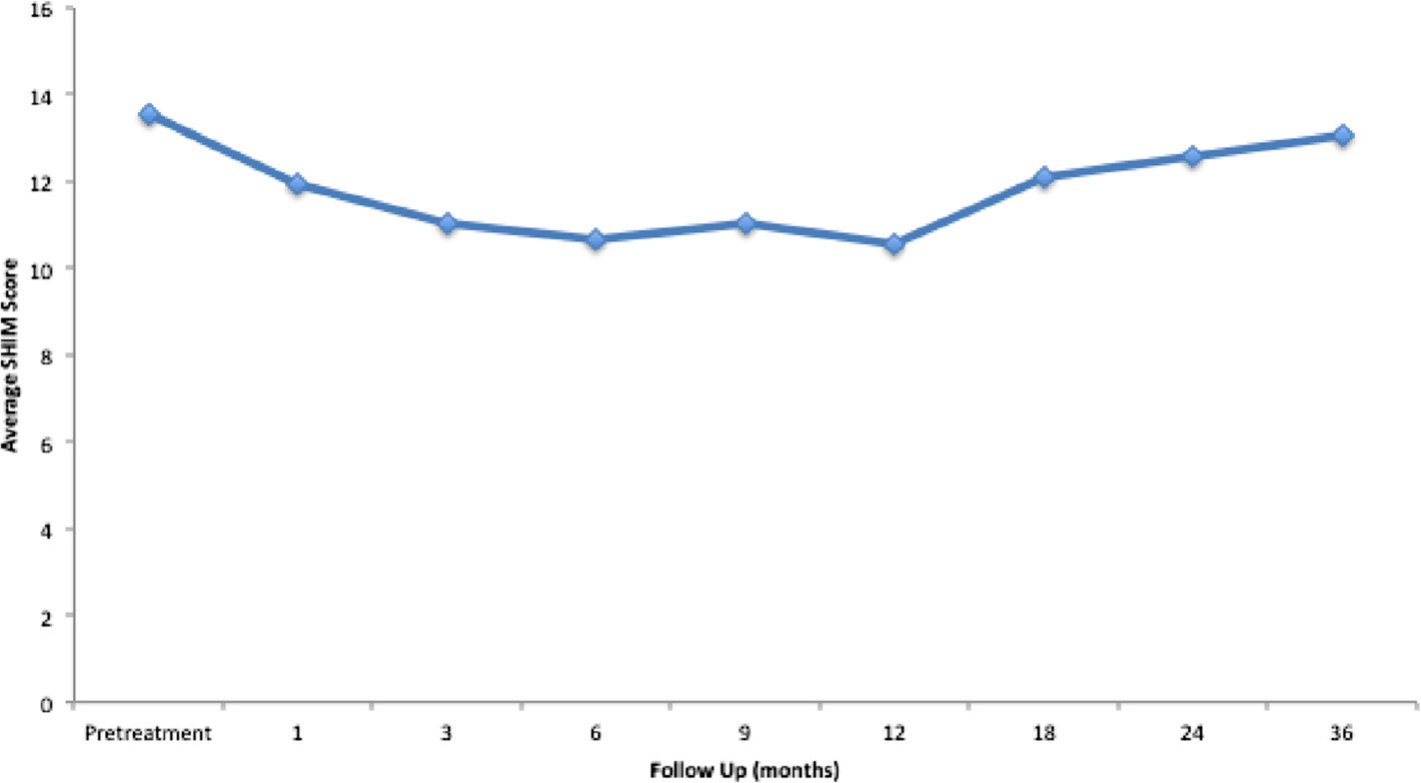
Mean SHIM scores following treatment up to 36 months of follow-up

Analysis of SHIM score was limited to patients who did not receive ADT (*n* = 92). The mean SHIM score prior to treatment was 13.52, which was consistent with mild to moderate erectile dysfunction (Table 3) (Fig. 4). SHIM score significantly decreased after 1 month to 11.95 (*p* < .001) and continued to decrease below baseline a year after treatment to 10.56 (*p* < .001). SHIM score began to improve at 18 months, but was still significantly less than baseline at 12.12 (*p* = .01). After 2 years, the mean SHIM score did not significantly differ from baseline at 12.57 (*p* = 0.34), and continued to improve after 3 years with a mean SHIM score of 13.06.

**Table 3:** Changes in mean SHIM score from baseline following SBRT for prostate cancer

	Pre	1 mo	3 mo	6 mo	9 mo	12 mo	18 mo	24 mo	36 mo
SHIM									
Mean	13.52	11.95	11.03	10.65	11.06	10.56	12.12	12.57	13.06
Mean difference		−1.57	−2.49	−2.87	−2.46	−2.96	−1.40	−0.95	−0.46
*p*-value		< .001	< .001	< .001	< .001	< .001	0.01	0.339	1

No patient reported RTOG Grade 3 or 4 urinary or rectal toxicity following treatment. The incidence of Grade two rectal toxicity was 3.0 % and the incidence of Grade two urinary toxicity was 9.9 % during follow-up. Complete obstruction, persistent hematuria, or persistent rectal bleeding was not observed in any patient during follow-up.

## Discussion

Post-treatment PSA response, sexual function, and urinary function all play an important role in the management of prostate patients following SBRT. The use of PSA as a surrogate endpoint is controversial. It has been suggested that a short post treatment PSA doubling time of less than 3 months is a good indicator of prostate cancer mortality after surgery or radiation therapy [32]. Similarly, an initial post treatment 3-month decline of 20 to 40 % has been shown to be a good indicator for overall survival [33]. This is accordance with our data which showed a 64 % PSA decline after 3-months. Since PSA expression is under hormonal control, androgen deprivation therapy can decrease PSA independent to tumor response. Because so few patients used ADT, 8.9 %, it is unlikely that this had a major effect on the data. Excluding ADT patients from the study did not significantly decrease biochemical free survival upon statistical analysis. Furthermore, our 3-year PSA level of 0.3 ng/ml shows a similar result to previously published data [19]. We expect the median PSA to further decrease with time, as Katz et al. reported a 0.1 ng/ml PSA level after 48 and 60 months following a similar treatment course [34]. Our data showed PSA bounces in 24 % of patients; this proved to be benign and transient as our 3-year biochemical failure-free survival rate was 100 %. This confirmed the excellent long-term disease control of SBRT previously demonstrated by Freeman et al. with a 5 year biochemical disease free survival of 93 % [25], and Chen et al. with a 99 % actuarial 2 year biochemical failure-free survival rate [29]. While our 5-year results are encouraging, 10–15 years of data is needed before definitive statements can be made on efficacy due to the long natural history of the disease. Long-term results should include data with 10+ years of results. The numbers presented in this paper can’t statistically signify dosimetric correlations and anomalies. While we believe doismetric parameters may effect short and long term toxicity, follow-up times are not long enough to do this statistical analysis.

Because the inclusion criteria for various modalities of treatment can differ, comparing therapeutic strategies is often imprecise [35], that being said, comparing the results of this study to other treatment modalities shows favorable results. Radiation has been shown to result in less obstructive and voiding issues when compared to surgery [36]. IPSS resolution following brachytherapy typically varies [27, 37], while IPSS scores, IPSS-I scores, and IPSS-O scores have been shown to return to baseline within 3 months following SBRT [20, 22, 38]. Our IPSS and IPSS-I results were similar to SBRT data showing a significant increase after 1 month and then no significant change from baseline after 3 months, however, IPSS-O scores did not significantly increase following treatment and actually decreased below baseline after 3 months. IPSS and IPSS-I also decreased below baseline after 2 years following the trend in previously published data [20, 22, 38].

Urinary flare is an established complication in literature following both SBRT and brachytherapy [39, 40]. This consists of urinary frequency, dysuria, and/or obstructive voiding systems lasting approximately 1 year after SBRT. This phenomenon is likely caused by bladder neck/urethral hyperemia in accordance with cystourethritis. Our data showed urinary flare after 1 year for total urinary symptoms and obstructive symptoms, and after 18 months for irritative symptoms.

Radiation therapy causes less voiding symptoms when compared to prostatectomy and this was demonstrated by our results. The trade-off to this decrease has been thought to be an increase in acute irritative symptoms following radiation and a decrease in irritative symptoms within 1 year of prostatectomy [41, 42]. The decrease in irritative symptoms following prostatectomy is thought to be from relief of prostatic obstruction. Relief of prostatic obstruction could also cause the reduction of symptoms in our data. SBRT is an ablative process and results in a decrease in prostate size within 3 years of treatment [43]; this decrease in size may result in improved urinary symptoms.

Erectile dysfunction (ED), defined as a SHIM score less than or equal to 21, has been reported to occur following both EBRT and prostatectomy. For either procedure, Potosky et al. reported a 75 % ED rate after 5 years [44], and Sanda et al. reported a 60 % ED rate after 2 years [28]. 76.3 % of our patients had some sort of baseline erectile dysfunction prior to treatment. Sexual potency, defined as a SHIM score less than or equal to ten, was maintained by the majority with 78 % of patients maintaining sexual potency 2 years after SBRT. Freeman et al*.* report that 82 % of patients who were sexually potent before treatment maintained erectile function post-treatment [16]. Furthermore, after 2 years, our data showed no significant change from baseline SHIM score and kept improving at 3 years. This was similar to previously reported data which showed a decline in SHIM after 1 year and then sexual function stabilization shortly after [29, 30].

There were several limitations to this study. The use of ADT results in reduction of prostate size, and could explain improvement in urinary symptoms following SBRT. This effect should be minimal as only 8.9 % of the patient population studied used ADT with a median of 4 months. The use of AUA and SHIM to assess function also narrows the scope of patient reported outcomes and should be explored further. The retrospective nature of this study carries biases. Carefully controlled prospective trial should be conducted to further confirm the effectiveness of SBRT in the treatment of prostate cancer.

## Conclusion

Prostate cancer patients treated with Cyberknife SBRT exhibited minimal acute toxicity. 3 year PSA response, reported toxicity, erectile function preservation, and urinary function improvement compares favorably to data presented following radical prostatectomy, brachytherapy, or conventional external beam radiation therapy. While an increase in AUA/IPSS score initially occurred, all patients resume normal activities immediately following treatment and the AUA/IPSS symptoms improved from baseline. Irittative symptoms take longer to resolve when compared to obstructive voiding symptoms in patients treated with SBRT.
